# A modal theory of discrimination

**DOI:** 10.1007/s11229-020-02747-4

**Published:** 2020-06-23

**Authors:** Guido Melchior

**Affiliations:** Department of Philosophy, University of Graz, Heinrichstrasse 26/5, 8010 Graz, Austria

**Keywords:** Sensitivity, Safety, Modal epistemology, Discrimination

## Abstract

Discrimination is a central epistemic capacity but typically, theories of discrimination only use discrimination as a vehicle for analyzing knowledge. This paper aims at developing a self-contained theory of discrimination. Internalist theories of discrimination fail since there is no compelling correlation between discriminatory capacities and experiences. Moreover, statistical reliabilist theories are also flawed. Only a modal theory of discrimination is promising. Versions of sensitivity and adherence that take particular alternatives into account provide necessary and sufficient conditions on discrimination. Safety in contrast is not sufficient for discrimination as there are cases of safety that are clearly instances of discrimination failure. The developed account of discrimination between objects will be extended to discrimination between kinds and between types.

## Introduction

We regularly employ discrimination when distinguishing between particular objects or persons, such as Anna from Hannah, between kinds or types, such as Ferraris from Lamborghinis, and between properties, such as cardinal red from carmine. So far, discrimination has been subject to philosophical reflection, but most, or even all, of these reflections do not primarily aim to develop a self-contained theory of discrimination. Rather, they utilize discrimination for explaining other philosophical concepts that are regarded as more interesting, in particular the concept of knowledge. These accounts discuss whether discrimination is a criterion for knowledge, often using a pre-theoretical concept of discrimination and, hence, leaving the concept of discrimination unexamined.

I think this is a methodological shortcoming. Discrimination is a well-known, common-sense concept and an epistemic theory of discrimination is desirable in its own right. Epistemic concepts other than knowledge are interesting apart from their relation to knowledge. Nevertheless, they are rather ignored in mainstream epistemology, though with some exceptions. Kvanvig (2003), for example, argues against the epistemic mainstream that understanding is the epistemic standing that is of crucial value and I advance in *Knowing and Checking: An Epistemological Investigation* (Melchior 2019) an account of checking, developing in a first step a modal theory of checking and using it in a second step to explain puzzles about knowledge.

In this paper, I will not contribute to the discussion on whether discrimination is necessary and/or sufficient for knowledge. Rather, I will develop a self-contained theory of discrimination.^1^ This theory is externalist in spirit—more precisely, it is a modal theory of discrimination. Since I remain neutral here about the connections between discrimination and knowledge, the externalist account of discrimination proposed here does not have any immediate impact on whether knowledge is externalist or internalist in nature. Nevertheless, the following results about discrimination and knowledge can be derived. If one opts for an internalist take on knowledge, then knowledge and discrimination obviously come apart due to the externalist nature of discrimination. However, even externalists about knowledge need not accept that knowledge and discrimination have identical modal conditions. For example, I will argue that versions of sensitivity and adherence are necessary and jointly sufficient for discrimination and that safety is necessary but not sufficient. Hence, a safety theorist about knowledge, who usually rejects sensitivity and adherence, can accept that there is a crucial difference between knowledge and discrimination.

In this paper, I will develop a sensitivity-based theory of discrimination. Sensitivity, as introduced by Nozick (1981), is a controversial condition on knowledge.^2^ Nevertheless, the principle is intuitively appealing and consequently there are different theories now on the market that utilize sensitivity in various contexts. Enoch et al. (2012) suggest that sensitivity is central for proof in court.^3^ Clarke-Doane and Baras (forthcoming) argue that sensitivity is necessary for undermining by defending the following principle of modal security: If evidence *e* undermines one’s belief that *p*, then *e* gives one reason to doubt that one’s belief is sensitive or safe. I develop in Melchior (2019) a sensitivity-based theory of checking. The theory provided here will argue in this sensitivity-friendly spirit that sensitivity is crucial for having the capacity to discriminate.

Let me briefly present two examples of epistemologists who refer to discrimination for developing a theory of knowledge or for solving particular knowledge puzzles: Goldman (1976) provides an externalist theory of perceptual knowledge that relies on discrimination. To a first approximation, Goldman (1976, p. 778) suggests that “S (noninferentially) perceptually knows that *p* if and only if (1) S (noninferentially) perceptually believes that *p*, (2) *p* is true, and (3) there is no relevant contrary *q* of *p* such that, if *q* were true (rather than *p*), then S would (still) believe that *p*.” In this definition, condition (3) is the discrimination condition. Goldman spells out discrimination in terms of counterfactual conditionals, as I will do in this paper. However, there are important differences between Goldman’s take on discrimination and the account that will be developed here. First, Goldman defines perceptual knowledge in terms of discrimination whereas this paper primarily focuses on discrimination. In this respect, Goldman’s approach is different in focus than the one presented here. Second, we will see that Goldman’s counterfactual condition (3) is not sufficient for discriminating between *p* and *q*. A stronger counterfactual conditional has to be fulfilled. Third, any appropriate account of discrimination requires conditions for discriminating between two particular alternatives whereas Goldman develops a theory of perceptual knowledge and consequently focuses on discriminating the target proposition *p* from any relevant alternative *q*. Finally, perception is a paradigmatic method of discrimination, but we will also consider non-perceptual instances of discrimination, for example discriminating even numbers from odd numbers.

Pritchard (2010, 2012) distinguishes between favoring and discriminating epistemic support and argues that a subject can have favoring epistemic support without having discriminating epistemic support. For example, S can have favoring epistemic support that the animal in the pen is a zebra and not a painted mule and that she is a normal person and not a brain in a vat, without having discriminating epistemic support. In his account, Pritchard relies on pre-theoretical intuitions about discrimination without providing a full theory.

Goldman emphasizes the connection between knowledge and discrimination by using a discrimination condition for developing a theory of perceptual knowledge. Pritchard, in contrast, utilizes a potential difference between knowledge and discrimination when arguing that a subject can know via favoring epistemic support although she lacks discriminatory support.^4^

In section two, I will make some general remarks on discrimination as a capacity and on particular instances of discrimination, setting the stage for a theory of discrimination. In section three, I develop a modal theory of discrimination. In section four, I extend this modal theory to reflective discrimination, discrimination via background knowledge, and discrimination of kinds and types.

## The capacity of discrimination

Actual instances of discrimination are manifestations of a discriminatory capacity that relies on the method used. For example, there is a difference between having the capacity to discriminate martins from blackbirds via eyesight and having this capacity by using an ornithological handbook. S might have the second capacity but not the first. It does not make sense to talk about discriminatory capacities simpliciter. Since there is no plausible way of talking about discrimination simpliciter, I will henceforth only consider discrimination via a particular method.^5^

Although discrimination always requires using a particular method, the method need not be *intentionally* used by the discriminating subject. For example, S can discriminate a martin from a blackbird via eyesight simply by seeing a martin and forming the belief that the bird is a martin. S need not raise the question whether a particular bird is a martin or a blackbird and to intentionally use a particular method for settling this question. For sure, discrimination can result from raising a question and intentionally using a method for settling this question, but this kind of inquiry is not necessary for discrimination.^6^

Discriminatory capacities always depend on particular environmental conditions. For example, S can have the capacity to discriminate Anna from Hannah via eyesight when it is sunny, but not when it is foggy. Moreover, capacities of discrimination can also depend on features of the subject, e.g. S can have the capacity to discriminate Anna from Hannah when S is sober but not when S is heavily drunk. Accordingly, we have to relativize discriminatory capacities to different conditions, environmental and subject-dependent. These conditions are not components of the method used, rather they are independent parameters. For example, we can distinguish the capacity to discriminate Anna from Hannah via eyesight under sunny conditions from the capacity to discriminate them via eyesight under foggy conditions. Often we talk about discriminatory capacities without mentioning particular conditions. In this case we assume that S has the discriminatory capacity under normal conditions, howsoever normal conditions might be spelled out in detail.^7^

We have to distinguish between the general capacity of discriminating *x* from *y* and actual instantiations of discrimination that manifest this capacity. If S actually discriminated *x* from *y* via method M then S has the capacity to discriminate *x* from *y* via M. However, the opposite entailment relation does not hold. S can have a general capacity to discriminate *x* from *y* even if she misidentified the target object in a particular case. In this case, no act of discrimination was performed although the person has the general capacity to discriminate.

Discrimination is symmetric, i.e. if S can discriminate *x* from *y*, then S can also discriminate *y* from *x*.^8^ For example, it is false to say that S can discriminate Anna from Hannah but she cannot discriminate Hannah from Anna.^9^ It might be subject of dispute whether actual discrimination is factive, i.e. whether the belief formed about *x* (or the belief formed about *y*) as a result of an act of discrimination is always true. Suppose, S has the general capacity of discriminating Anna from Hannah, but in a particular case S forms the false belief that the target person is Anna. S manifested a discriminatory capacity, but did S actually discriminate Anna from Hannah? To my lights, an affirmative answer is implausible. Consequently, I will hereinafter assume that actual discrimination is factive, i.e. S discriminated *x* from *y* if and only if S forms a *true* belief that the target object is *x* (or that it is *y*) and the belief formation manifests S’s capacity to discriminate *x* from *y*. Accordingly, I will develop here a modal theory of factive discrimination. However, one might not share the intuition that acts of discrimination are always factive. In this case, the presented theory can be modified and extended to cover non-factive discrimination.^10^

Discrimination applies to different types of entities. First, we can discriminate between particular objects, for example I can discriminate Anna from her twin sister Hannah. Second, we can discriminate between certain properties and kinds and types of objects, for example if S can discriminate Ferraris from Lamborghinis.^11^ Third, we can discriminate between certain situations or states of affairs, for example when I can discriminate being drunk from having circulatory system problems. Such talk about discriminating between certain situations or state of affairs is also involved when claiming that we cannot discriminate between being a real person in a normal world and being a completely deceived brain in a vat.

We already saw that we can discriminate different entities, such as objects and persons, properties, types, and kinds. When discriminating between two objects *x* and *y*, the resulting proposition is about an object being *x* (or being *y*), e.g. ‘This is Anna.’ As for properties, kinds and types, we usually use phrases such as ‘This is an F’; e.g. when saying that this car is a Ferrari. In any of these cases, we need not refer to the target object via a demonstrative. We can also use a definite description, e.g. when saying that the person in the garden is Anna.

Discriminating *x* from *y* typically involves a resulting belief that the target object is *x* (or that it is *y*) or that it is an F (or that it is a G).^12^ However, discrimination need not necessarily lead to beliefs. For example, some basic acts of perceptual discrimination in animals or even humans plausibly do not. Moreover, there are cases in which we ascribe a discriminatory capacity to persons if they do not form full beliefs about what the target objects are. Suppose that elms and beeches look differently to S such that S can separate them reliably into two groups and articulate that these are trees of two different kinds, but without knowing that they are elms and beeches. There is a sense in which we can say that S can discriminate elms from beeches although S does not form any true de dicto beliefs about elms and beeches. These instances of discrimination might be classified as incomplete or de re instances of discrimination. In this paper, I am only interested in complete discrimination leading to de dicto beliefs about the items discriminated. Thus, I will provide a modal theory for belief-forming instances of discrimination, which I regard as paradigmatic cases of full-fleshed discrimination. Any references to discrimination hereinafter are meant to capture complete de dicto discrimination.

Let me briefly reflect on the relation between discrimination and ruling out (relevant) alternatives. Some theories of knowledge have it that, in order to know that *p*, a subject has to rule out certain alternatives where alternatives are propositions that are incompatible with *p*. Usually these theories accept that not every proposition that is incompatible with *p* must be ruled out for knowing that *p*, but only every relevant alternative.^13^ One might suspect that there is a strong connection between discrimination and ruling out alternatives. For example, there is an intuitive problem of discriminating zebras from painted mules via eyesight and there is an analogous problem of ruling out via eyesight that the animal that looks like a zebra is not a painted mule. Moreover, there is an intuitive problem of discriminating being in a normal world from being in a skeptical deception scenario and there is an analogous problem of ruling out skeptical hypotheses. Thus, one might be tempted to argue that ruling out an alternative for *p* requires having a corresponding discriminatory capacity, i.e. S can rule out an alternative *q* for *p* only if S has the capacity to discriminate between *p* and *q*.

Indeed, in deception cases, that involve phenomena such as painted mules or skeptical scenarios, failure to rule out alternatives goes hand in hand with a lack of discriminability. However, it is not clear whether this connection can be established for other cases, in particular, cases involving lottery propositions. For example, there is an intuitive problem of knowing that my car is still parked in lot 66 if I cannot rule out the alternative that it has been stolen 5 min ago. Moreover, there is a problem of knowing that I will not have enough money for going on a safari if I cannot rule out the alternative that I will win the lottery.^14^ However, it is doubtful that there is an analogous issue of discriminating between my car still being parked in lot 66, where I parked it 2 h ago, and having been stolen 5 min ago and whether there is an issue of discrimination in the safari case. Perhaps, one thinks that the car-case can be paraphrased as discrimination (or lack of discrimination) between cars that are still parked in lot 66 and cars that have been stolen, but I do not see how we can correctly say that one can discriminate not having enough money for going on a safari from winning the lottery. Hence, there are certain issues concerning ruling out alternatives that do not rely on more fundamental issues concerning discrimination. Therefore, these phenomena must be kept apart. This does not affect the aim of this paper of developing a theory of discrimination. However, it is worth noting that general relevant alternative theories must be developed independently of discrimination, whatever such accounts might look like.^15^

Perception is a paradigmatic method of discrimination.^16^ Accordingly, paradigmatic instances of discrimination involve two objects, properties, or types of objects that *look or seem* to a subject in a particular way. For example, non-trivial and often cited cases of discrimination involve twin-siblings that look very similar or two very similar colors that can be discriminated by an expert but not by a layperson. Moreover, we intuitively affirm that we cannot discriminate being a normal person from being a brain in a vat because in both cases ex hypothesi everything seems to us in the same way. One might suspect that discrimination crucially involves objects, properties, or scenarios looking or seeming in a certain way to us. If this view were correct, then an internalist theory of discrimination were promising which has it that the capacity of discrimination always depends on how the target objects, properties, or facts look or seem to the subject. However, this view is mistaken. For example, S can lack a de dicto capacity to discriminate elms from beeches because she lacks knowledge about the characteristic features of each kind although elms and beeches do not look to S in the same way. Moreover, we reasonably talk about discrimination in cases that do not involve any seeming. For example, we can correctly say that a subject can discriminate odd numbers from even numbers by checking whether the last number is a 0, 2, 4, 6, 8 or that she can discriminate inductive arguments from abductive arguments although no seeming is involved in these cases.^17^ Since there are cases where subjects lack a full, de dicto discriminatory capacity despite distinct seemings and there are cases of discrimination without seemings, an internalist theory of discrimination based on seeming must fail. This motivates the following development of an externalist theory of discrimination.

Schaffer (2004b, p. 150) develops an experience-based account of discrimination. He defines that “S has the capacity to discriminate proposition *p* from proposition *q* iff (1) *p* and *q* are exclusive, (2) S’s experience is compatible with *p*, and (3) S’s experience is incompatible with *q*. And S discriminates *p* from *q* (exercises the capacity) iff S believes that *p* rather than *q* on the basis of S’s capacity to discriminate *p* from *q*.” According to Schaffer (2004b), since discrimination contains an experiential and a doxastic element, there are three ways a subject can fail to discriminate *p* from *q*: “(1) one can lack the requisite experience (blindness), (2) one can lack the requisite beliefs (ignorance), or (3) one can have the requisite experience and the beliefs but fail the basing relation (luck)” (p. 151). Schaffer adopts Dretske’s zebra case to illustrate his point. Schaffer argues that a layperson who cannot tell a zebra from a painted mule via eyesight and an expert who can tell them apart both have the requisite experience to discriminate zebras from painted mules, since, by assumption, painted mules look slightly different than zebras. However, they differ in their capacity to form correct beliefs based on their experiences. The layperson fails to discriminate zebras from painted mules due to ignorance since she lacks the requisite belief whereas the expert succeeds in discrimination.

We have already seen that there are plausible instances of discrimination that do not involve any experiences. Hence, Schaffer’s conception of discrimination is too narrow. Moreover, his account has a further counterintuitive consequence. According to his account, the layperson and the expert both have the capacity to discriminate zebras from painted mules, but only the expert can exercise this capacity. This is implausible. It seems false to ascribe a full capacity to discriminate *x* from *y* to someone who cannot form any true beliefs about the target object being *x* or being *y*. Rather, such persons lack this discriminatory capacity, at least the capacity of full-fleshed discrimination involving de-dicto beliefs, which is subject of this paper. More plausibly, only the expert but not the layperson has the capacity to discriminate. This is precisely the result of the discrimination account developed here that relies on doxastic but not on experiential factors.

## Discrimination and modality

In this section, I will develop a modal account of discrimination. This account has it that a version of sensitivity that takes particular alternatives into account and that is stronger than ordinary sensitivity is necessary for discrimination. Moreover, we will see why an analogously modified safety condition is not sufficient. In the first subsection, I present paradigmatic instances of discrimination and briefly review the modal epistemic conditions of sensitivity, adherence, and safety. In the second subsection, I formulate versions of these modal conditions that take specific alternatives into account, which are crucial for a modal theory of discrimination. I argue in the third subsection that the modal structure of discrimination cannot be spelled out in terms of safety, since paradigmatic instances of discrimination failure are safe. In the last subsection, I present a modal account of discrimination based on sensitivity and adherence concerning specific alternatives, briefly reflect on the connections to other epistemic theories that utilize the sensitivity principle, and discuss to what extent the provided account of discrimination faces the generality problem.

### Paradigmatic discrimination and modal conditions

What do we mean when we say that S can discriminate *x* from *y* via a particular method, for example if S can discriminate Anna from Hannah via eyesight? What we paradigmatically mean is something along the following lines: S believes via eyesight that the person in question is Anna if it is Anna and that she believes via eyesight that it is Hannah if it is Hannah. This formulation allows for different externalist interpretations. First, we can opt for a statistical interpretation. In this case, the statistical frequency of S’s correct identifications via eyesight determines whether S has the capacity to discriminate Anna from Hannah. If S forms accurate beliefs via eyesight above a certain threshold, perhaps 80%, about the target person being Anna (or being Hannah), then S can discriminate Anna from Hannah; otherwise she cannot.

However, discriminatory capacities are dispositional. S can have the capacity to discriminate *x* from *y* even if she has never applied this capacity. For example, I have the capacity to discriminate Ferraris from Space Shuttles although I have never actually seen a Space Shuttle and therefore never actually applied this capacity. However, a statistical reading of discrimination requires that S has a discriminatory capacity only if a certain statistical threshold is exceeded which is the case only if S has accomplished actual acts of discrimination numerous times. Thus, discrimination has to be spelled out in terms other than statistical ones. More properly, discrimination is formulated in modal terms that not only consider actual instances of discrimination but possible ones as well. A paradigmatic discriminatory capacity can be modally characterized as follows:

**Paradigmatic discrimination**

S can paradigmatically discriminate between *x* and *y* via M *iff*

- (D1) In the nearest possible worlds where the target object is *x* and where S uses M, S believes via M that it is *x* (and not *y*).^18^
- (D2) In the nearest possible worlds where the target object is *y* and where S uses M, S believes via M that it is *y* (and not *x*).

We already saw that discriminatory capacities can be relativized to particular conditions, environmental or subject-dependent. Accordingly, we can characterize paradigmatic discrimination *given that particular conditions are fulfilled* as follows:

**Paradigmatic discrimination under conditions C**

S can paradigmatically discriminate between *x* and *y* via M given that conditions C are fulfilled *iff*

- (D1) In the nearest possible worlds where the target object is *x* and where S uses M and where C are fulfilled, S believes via M that it is *x* (and not *y*).
- (D2) In the nearest possible worlds where the target object is *y* and where S uses M and where C are fulfilled, S believes via M that it is *y* (and not *x*).

In the following, I will ignore specific conditions if they are not crucial for making my point. Since we are now talking about modal epistemic conditions, let me, at this point, provide a brief overview of existing modal conditions for *knowledge*, which I will then modify for discrimination. Nozick (1981) argues, to a first approximation, that S knows that *p* iff (1) S believes that *p,* (2) *p* is true, (3) in the nearest possible worlds where *p* is false S does not believe that *p*, and (4) in the nearest possible worlds where *p* is true S believes that *p*. Conditions (3) and (4) are modal conditions. (3) has become known as the sensitivity condition and (4) as the adherence condition.^19^ Nozick realized that his knowledge account is inadequate if it does not take the belief forming method into account, and so he suggests that S knows that *p* via method M iff S’s belief formed via M is true and the following two conditions are fulfilled:

- **Method-relative sensitivity**
  In the nearest possible worlds where *p* is false and where S uses M to determine whether *p* (or ~ *p*) is true, S does not believe via M that *p*.
- **Method-relative adherence**
  In the nearest possible worlds where *p* is true and where S uses M to determine whether *p* (or ~ *p*) is true, S believes via M that *p*.^20^

Nozick’s sensitivity account faces well known problems. In particular there are plausible instances of inductive knowledge that is insensitive as Vogel (1987) and Sosa (1999) point out, and Nozick’s account also implies implausible instances of closure failure as Kripke (2011) argues.^21^ Given these problems, sensitivity is plausibly an inadequate condition on knowledge, but we will see that a modified version is crucial for discrimination.

As a reaction to these problems, Sosa (1999) suggests replacing Nozick’s sensitivity condition with a safety condition. S’s belief that *p* is safe iff in the nearest possible worlds where S believes that *p*, *p* is true. Sosa claims that S knows that *p* only if S truly and safely believes that *p*. As for sensitivity and adherence, we can formulate a method-relative version of safety:

**Method-relative safety**

In the nearest possible worlds where S believes that *p* via M, *p* is true.^22^

### Modal conditions and alternatives

Modal conditions on knowledge are specified in terms of *p*-worlds or ~ *p*-worlds without stipulating exactly how the ~ *p*-worlds are configured. With discrimination the situation is different. For determining whether S can discriminate *x* from *y*, we do not just consider possible worlds where the target object is not *x*. The possible worlds we consider must be those where the target object is *y* and not *x*, i.e. we consider particular *y*-alternatives that are incompatible with the target object being *x*. This fact has to be taken into account when spelling out modal conditions on discrimination. In order to determine whether S can discriminate *x* from *y* via M, we not only consider what S would believe via M if the target object where not *x*. Rather we consider what S would believe via M *if it were y* and not *x*. For example, for determining whether S can discriminate Anna from Hannah via eyesight, we consider what S would believe via eyesight if the target person were Hannah and not Anna. Scenarios where she is Claudia and not Anna are not relevant for determining S’s capacity to discriminate Anna from Hannah.

Accordingly, a discrimination-relevant sensitivity condition not only requires that S does not believe that the target object is *x* via M in the nearest possible worlds where it is not *x*. Rather it requires that S does not believe that it is *x* via M in the nearest possible worlds where it is *y* and not *x*. Let us call this version of sensitivity *alternative*-*sensitivity* or *a*-*sensitivity* in brief. For reasons that will become obvious soon, I call the following version of sensitivity *weak* a-sensitivity:

**Weak a-sensitivity**

In the nearest possible worlds where the target object is *y* and where S uses M, S does not believe via M that it is *x*.

Weak a-sensitivity has always to be formulated for two particular alternatives *x* and *y*, in contrast to ordinary sensitivity. Weak a-sensitivity concerning a particular alternative can be stronger than ordinary sensitivity but it can also be weaker depending on how the cases are described. Take first a version of Dretske’s (1970) zebra case and suppose that S is standing in front of a zebra in a trustworthy zoo that normally does not exhibit faked animals. In the nearest possible worlds where there is no zebra in the pen a different animal such as a lion (or no animal) is in the pen and S does not believe that there is a zebra in the pen. Hence, S’s belief that there is a zebra in the pen fulfills ordinary sensitivity. However, S’s belief is not weakly a-sensitive with respect to the particular alternative that there is not a painted mule in the pen, since in the nearest possible worlds where there is a painted mule in the pen instead of a zebra, S believes that there is a zebra in the pen. In this case, weak a-sensitivity for there being a zebra in the pen and not a painted mule is a stronger condition than ordinary sensitivity for there being a zebra in the pen.

Now take a different case: Suppose S is standing in front of a pen in a different zoo that is, unbeknownst to S, not trustworthy. Many objects that look like animals in the pen are actually made of papier-mâché. In the nearest possible worlds where there is no zebra in the pen, a faked papier-mâché zebra is in the pen. In these possible worlds S’s belief that there is a zebra in the pen fails to fulfill ordinary sensitivity. Suppose further that the director of the zoo is a passionate painter and thinks that painted animals but not papier-mâché animals are objects of art that are worth being exhibited in the zoo. Thus, in the nearest possible worlds where there is a painted mule in the pen instead of a zebra, the zoo indicates that there is a painted mule in the pen and, consequently, S does not believe that there is a zebra in the pen. Thus, S’s belief that there is a zebra in the pen fulfills weak a-sensitivity. In this case, ordinary sensitivity for there being a zebra in the pen is stronger than weak a-sensitivity for there being a zebra in the pen and not a painted mule.

However, weak a-sensitivity is a condition too weak for being sufficient for discrimination. Suppose S believes that the target object is *x* via eyesight, but S does not even know that *y* exists and does not possess any concept of *y* such that in the nearest possible worlds where S is confronted with *y*, S does not form any belief that the target object is *y*. In this case, S plausibly cannot discriminate *x* from *y* since no general capacity of discrimination is manifested. For example, S cannot discriminate Ferraris from Lamborghinis if S does not know that Lamborghinis exist and does not possess any concept of them.^23^ Moreover, discrimination is symmetric, i.e. if S can discriminate *x* from *y*, then S can also discriminate *y* from *x*. However, it is even more implausible to say that S has a capacity to discriminate *y* from *x* if S does not know that *y* exists and never forms a belief that the target object is *y*.^24^ However, weak a-sensitivity via eyesight is fulfilled, since in the nearest possible worlds where the target object is *y* and not *x* and where S uses eyesight, S does not believe that it is *x*. Hence, weak a-sensitivity concerning *x* and *y* is not sufficient for discriminating *x* from *y*.

In this particular case, discrimination fails because S never believes that the target object is *y* if it is *y*, although S does not believe that it is *x* if it is *y*. These cases can be excluded by strengthening a-sensitivity as follows:

- **Strong a-sensitivity**
- In the nearest possible worlds where the target object is *y* and where S uses M, S believes via M that it is *y*.

This version of a-sensitivity is stronger because believing that the target object is *y* implies not believing that it is *x*, but not vice versa.^25^ According to the original formulation of sensitivity, we consider the nearest ~ *p*-worlds for determining whether S’s belief that *p* is sensitive. This formulation does not work for discrimination since we have to consider more specific ~ *p*-possible worlds where the target object is *y* instead of *x*.^26^

Goldman’s (1976) theory of perceptual knowledge relies on the discrimination condition that there is no relevant contrary *q* of *p* such that, if *q* were true (rather than *p*), then S would (still) believe that *p*. His theory of perceptual knowledge via discrimination is based on a version of weak a-sensitivity. While this condition might be sufficient for explaining perceptual knowledge in terms of discrimination, it does not suffice for a general theory of discriminating *p* from any relevant contrary *q*. Such a theory has to contain the following version of strong a-sensitivity: For every relevant contrary *q* of *p*, if *q* were true (rather than *p*), then S would believe that *q* (rather than *p*). Hence, Goldman’s theory of perceptual knowledge is not based on a general theory of discriminating *p* from any relevant contrary *q*.

In contrast to sensitivity, the standard formulations of adherence and safety only mention worlds where *p* is true but not worlds where *p* is false. Hence, we cannot reformulate adherence and safety analogously to sensitivity by talking about *y*-worlds instead of more generally ~ *p*-worlds. Accordingly, we can use the following ordinary versions of adherence and safety also for discrimination. Again, for reasons that will become obvious soon, I call these versions strong a-adherence and weak a-safety:

- **Strong a-adherence**
- In the nearest possible worlds where the target object is *x* and where S uses M, S believes via M that it is *x*.
- **Weak a-safety**
- In the nearest possible worlds where S believes via M that the target object is *x*, it is *x*.^27^

We distinguished weak from strong a-sensitivity. Weak a-sensitivity requires that in the nearest *y*-worlds, S does not believe that the target object is *x*, whereas strong a-sensitivity requires that in the nearest *y*-worlds, S believes that it is *y*. Moreover, a-adherence and a-safety can be modified in an analogous way. By replacing ‘S believes via M that the target object is *x*’ by ‘S does not believe that the target object is *y*’ we can formulate a weak version of a-adherence and a strong version of a-safety:

- **Weak a-adherence**
- In the nearest possible worlds where the target object is *x* and where S uses M, S does not believe via M that it is *y*.
- **Strong a-safety**
- In the nearest possible worlds where S does not believe via M that the target object is *y*, it is *x*.^28^

### Discrimination and safety

I will argue that strong a-sensitivity and strong a-adherence for *x* and *y* are reasonably necessary and sufficient for discrimination. Before making this point, let me focus on safety, currently the most popular modal condition on knowledge. By reflecting on safety, we will see that safety does not capture the modal features of discrimination since some paradigmatic instances of discrimination failure fulfill weak a-safety. This suggests that the modal conditions on knowledge and discrimination come apart.

Strong a-safety is obviously not necessary for discrimination. Suppose that S can perfectly discriminate between *x* and *y* via M. Suppose further that there are many nearby possible worlds where the target object is *z* and that in these possible worlds S believes via M that the target object is *z*. In this case, strong a-safety is violated since there are many nearby possible worlds where S does not believe via M that the object in question *y*, although it is not *x*. Nevertheless, S can perfectly discriminate between *x* and *y*. Therefore, we can ignore strong a-safety from now on.

Weak a-safety is plausibly necessary for discrimination. If, in many nearby possible worlds where S believes via M that the target object is *x*, it is *y*, then plausibly S cannot discriminate *x* from *y*. For example, if there are many nearby possible worlds where S believes via eyesight that the person in question is Anna when in fact it is Hannah, then S cannot discriminate Anna from Hannah.

Let us now reflect on whether weak a-safety is sufficient for discrimination. Let me present three paradigmatic instances of discrimination failure– random beliefs, opposing beliefs, and monotonous beliefs. We will see that weak a-safety can be fulfilled in the case of monotonous beliefs, and so we can conclude that weak a-safety is not sufficient for discrimination. Here is a first case of discrimination failure:

**1. Random beliefs**

S randomly forms beliefs via M about whether the target object is *x* or *y*.

If S randomly forms beliefs about whether the target object is *x* or *y*, then there is no connection between the status of the target object being *x* (or being *y*) and what S believes about it. Consequently, in many nearby possible worlds where the target object is *x*, S believes that it is *y* and vice versa. In this case, S obviously does not have the capacity to discriminate *x* from *y*. Example: S cannot discriminate Anna from Hannah via eyesight if S randomly forms beliefs via eyesight about whether the person in question is Anna or Hannah. Let me now come to a second type of paradigmatic discrimination failure:

**2. Opposing beliefs**

- S always forms false beliefs via M about whether the target object is *x* or *y*.
- In the nearest possible worlds where the target object is *x* and where S uses M, S believes via M that it is *y*, and in the nearest possible worlds where the target object is *y* and where S uses M, S believes via M that it is *x*.

With opposing beliefs, S has some capacity to distinguish between *x* and *y*, but S still fails to have a discriminatory capacity that reliably yields true beliefs. Example: S cannot discriminate Anna from Hannah via eyesight if S believes via eyesight that the person in question is Hannah if she is Anna and believes that she is Anna if she is Hannah.^29^

Random beliefs and opposing beliefs violate weak a-safety. In the case of random beliefs, there are many nearby possible worlds where S believes that the target object is *x* although it is *y*. As for opposing beliefs, *all* nearby possible worlds where S believes that the target object is *x* are such that it is *y*. Thus, these two instances of discrimination failure do not pose a problem for a potential safety account of discrimination. However, let us consider a third case of paradigmatic discrimination failure, one where weak a-safety can be fulfilled:

**3.Monotonous beliefs**

- S always forms the same belief via M about whether the target object is *x* or *y* regardless of whether it is *x* or *y*.
- In the nearest possible worlds where the target object is *x* and where S uses M, S believes via M that it is *x*, and, in the nearest possible worlds where the target object is *y* and where S uses M, S believes via M that it is *x*.

Discrimination requires responsiveness to the world, i.e. S can discriminate *x* from *y* via M only if S’s beliefs about *x* and *y* are in some way responsive to the facts involving *x* or *y*. Monotonous beliefs lack this kind of responsiveness to the facts and, therefore, do not manifest any discriminatory capacity. If S always believes that the target object is *x*, regardless of whether it is *x* or *y*, then S is simply not responsive to the world concerning *x* or *y*. Example: S cannot discriminate Anna from Hannah via eyesight if S always believes via eyesight that the person in question is Anna regardless of whether she is Anna or Hannah, e.g. because S does not even know that Hannah exists.

Random and opposing beliefs do not fulfill weak a-safety. Monotonous beliefs, in contrast, can be weakly a-safe. Suppose that S has a monotonous belief that the target object is *x*. Suppose further that for some reason any possible world where the target object is *y* is remote. In this case, weak a-safety is still fulfilled. In the nearest possible worlds where S believes that the target object is *x*, it is *x*, since there simply are no nearest possible worlds where the target object is not *x* (but *y*). Take the example of two twin sisters Anna and Hannah. Suppose that Anna lives next door to S but that Hannah lives far away from Anna and never visits her. Hence, any possible world where S sees Hannah is modally remote. Suppose further that S knows Anna but that S does not know that Hannah exists. Weak a-safety is fulfilled simply because any world where S sees Hannah is modally remote.^30^ However, in the nearest possible worlds where the person in question is Anna and in the nearest possible worlds where it is Hannah, S believes that it is Anna. Therefore, S’s belief that the person in question is Anna is monotonous. Due to this monotonicity, S cannot discriminate Anna from Hannah. Thus, despite fulfillment of weak a-safety, S cannot discriminate Anna from Hannah. Hence, weak a-safety is not sufficient for discrimination.

### Sensitivity, adherence, and discrimination

Let me recall the two jointly necessary and sufficient conditions for paradigmatic discrimination:

(D1): In the nearest possible worlds where the target object is *x* and where S uses M, S believes via M that it is *x* (and not *y*).
(D2): In the nearest possible worlds where the target object is *y* and S where uses M, S believes via M that it is *y* (and not *x*).^31^

(D1) and (D2) are versions of strong a-sensitivity and strong a-adherence. They can be labelled in different ways. From the standpoint of *x* being the case and *y* being the alternative, (D1) is strong a-adherence and (D2) is strong a-sensitivity. Inversely, from the standpoint of *y* being the case and *x* being the alternative, (D1) is strong a-sensitivity and (D2) is strong a-adherence. Discrimination is a symmetric capacity. S can discriminate *x* from *y* iff S can discriminate *y* from *x*. Consequently, adequate analyses and descriptions of discriminatory capacities are also symmetric. Hence, an analysis of discrimination does not favor any of the two standpoints. Both descriptions are equally adequate for this purpose.^32^

(D1) and (D2) are jointly sufficient for *paradigmatic* discrimination. Moreover, weak versions of sensitivity and adherence are not sufficient as we have seen. Furthermore, discrimination is symmetric, i.e. S can discriminate *x* from *y* iff S can discriminate *y* from *x*. Hence, there is no modal condition that has to be fulfilled for *x* which need not be fulfilled for *y*. Thus, if (D1) is necessary for discrimination, then (D2) is also necessary. We can reasonably assume at this point that fulfilling conditions (D1) *and* (D2) are both necessary, and jointly sufficient for discrimination. The huge variety of potential modal conditions on discriminating *x* from *y* boils down to these two conditions that are jointly necessary and sufficient.^33^

The formulation of “nearest possible worlds” in (D1) and (D2) is vague. Hence, these conditions can come in different strengths. The larger the neighborhood of possible worlds is where S has to form true beliefs about the target object being *x* (or being *y*), the stronger the conditions are. Moreover, (D1) and (D2) can come in different strengths depending on whether S has to form true beliefs in *all* or only in *most* nearby possible worlds (and depending on the standards for ‘most’.) In this paper, I will not offer an ultimate definition of discrimination answering all these questions. Since discriminatory capacities come in degrees such an ultimate definition is not required. However, at least some version of (D1) and (D2) is necessary for discrimination.

We have already seen that weak a-safety is not sufficient for discrimination because monotonous beliefs can be weakly a-safe. If S’s belief that the target object is *x* is monotonous, then in the nearest possible worlds where it is *y* S believes that it is *x*. Hence, strong a-*sensitivity* (from the standpoint of x being the case) is violated. Monotonicity of beliefs does not rule out weak a-safety, but it is incompatible with strong a-sensitivity (and strong a-adherence). Thus, a sensitivity account of discrimination can explain discrimination failure in case of monotonous beliefs whereas a safety account cannot. Moreover, also random beliefs and opposing beliefs fail to fulfill strong a-sensitivity. Hence, a sensitivity account of discrimination delivers the desired result that discrimination is incompatible with randomness, opposition, and monotonicity. Sensitivity and adherence are rather implausible and unpopular conditions on knowledge. However, they are crucial for discrimination. This supports the view that there is a crucial conceptual difference between knowledge and discrimination.

At this point, one might wonder what the connections are between discrimination and other epistemic phenomena that are said to involve sensitivity such as checking (Melchior 2019), proof in court (Enoch, Fisher, and Spectre 2012) or undermining (Clarke‐Doane and Baras forthcoming). I argue in *Knowing and Checking: An Epistemological Investigation* (Melchior 2019) that successfully checking whether *p* requires intentionally using a method which is sensitive with respect to *p*. Discrimination, in contrast, can happen unintentionally and requires a-sensitivity concerning a specific alternative, not sensitivity simpliciter.^34^ One might argue that, if proving in court requires sensitivity as Enoch, Fisher, and Spectre (2012) suggest, then it is an instance of checking. If this assumption is correct, then there are similar analogies and differences between discrimination and proving in court as there are between discrimination and checking.^35^

Clarke‐Doane and Baras (forthcoming) defend the principle of modal security that, if evidence *e* undermines one’s belief that *p*, then *e* gives one reason to doubt that one’s belief is sensitive or safe. We saw that a-sensitivity and a-safety are necessary for discrimination. One can argue analogously that if *e* is evidence that one cannot discriminate *x* from *y* via method M then *e* gives reason to doubt that M is a-safe or a-sensitive with respect to *x* and *y*.^36^ In this respect, the principle of modal security can be adapted for discrimination. However, we cannot directly maintain that evidence *e* undermines one’s belief iff *e* undermines one’s capacity to discriminate. First, not every belief results from exercising a discriminatory capacity. Hence, there are beliefs that are undermined by evidence but there is no corresponding discriminatory capacity that is analogously undermined. Second, undermining a belief involves safety or sensitivity simpliciter whereas undermining of discrimination always involves a-sensitivity and a-safety concerning a particular alternative. We have seen that sensitivity simpliciter does not entail a-sensitivity and vice versa (and the same also holds for safety). Hence, there can be evidence that undermines one’s belief but not one’s capacity to discriminate and vice versa.

Externalist knowledge accounts suffer from the notorious generality problem.^37^ This problem was originally formulated for process reliabilism, but any other externalist knowledge account that relies on specific methods used, in particular modal knowledge accounts that are based on method-relative sensitivity or method-relative safety, are also affected by this problem.^38^ Suppose S observes a car at night under a lantern and forms the belief that the car is blue. In order to decide whether the belief is reliably formed, we have to determine what the respective belief forming method is, e.g. whether it is eyesight simpliciter, eyesight at night, eyesight at night under a lantern, and so on. The modal account of discrimination proposed here only partly suffers from the generality problem. S only possesses discriminatory capacities with respect to a particular method. There is no discriminatory capacity simpliciter. For example, S might have the capacity to discriminate blue cars from grey cars via eyesight and via eyesight at night under a lantern but not via eyesight at night.^39^ However, it does not make sense to ascribe discriminatory capacities simpliciter without referring to a particular method, at least implicitly. In this respect, there is no generality problem for discriminatory *capacities*.^40^ In contrast, we ascribe knowledge and justification simpliciter, not via a particular method.^41^ Therefore, this circumvention of the generality problem is not available for knowing and justified believing.^42^

The situation is different for particular instances of discrimination, which are always a manifestation of a discriminatory capacity. A belief that a target object is *x* (or that it is *y*) can manifest different capacities. In order to determine whether S actually discriminated *x* from *y*, we have to determine which capacity the discriminatory act manifests. Here we face the generality problem. In this respect, the generality problem affects determination of particular instances of discrimination but not determination of general discriminatory capacities.

## Extensions: reflective discrimination, background knowledge, and discrimination of kinds and types

So far, we have focused on discriminating between particular objects. In this section, I will sketch how the sensitivity account of discrimination can be extended in various ways. Any act of discrimination involves a particular method. We have seen that discrimination is not intentional in that the discriminating subject need not use this method with the particular intention of discriminating the target object from specific alternatives. Rather, subjects can form beliefs unintentionally via discrimination methods, for example if S comes to believe that the bird in her garden is a blackbird and not a martin when accidentally looking out of the window. Moreover, discrimination need not be *reflective* in the sense that the discriminating subject knows that the belief formed via a particular method has those modal features that are required for discrimination. For example, an infant can discriminate blue balls from red balls without knowing that she has this capacity. Nevertheless, a subject that can discriminate *x* from *y* via method M and knows that beliefs formed via M have those modal features required for discrimination is in a better epistemic position than a subject that has this discriminatory capacity but lacks this kind of knowledge.^43^ In order to do justice to this differentiation, I will call discrimination that involves knowledge about the modal features of the method used *reflective discrimination*.

A general capacity of discriminating *x* from *y* has to be distinguished from the more specific capacity of discriminating *x* from *y given that S has background knowledge that the target object is either x or y*. This is, for example, the case when S knows that the person in front of her is either Anna or Lydia, and based on this background knowledge S has to determine whether it is Anna or Lydia. The specific capacity of discriminating *x* from *y* based on such background information does not entail a general capacity to discriminate *x* from *y*. For example, without any background knowledge S believes that the person in Claudia’s kitchen who she sees through the window across the street is Claudia because S knows that it is Claudia’s apartment even though the person she sees is Anna or Lydia. However, given that S knows that it is either Anna or Lydia, S believes that it is Anna if it is Anna and believes that it is Lydia if it Lydia. In this case, S lacks a general capacity to discriminate Anna from Lydia via looking into Claudia’s kitchen, but S has this capacity given that she has background knowledge that it is either Anna or Lydia. In contrast, if S has the general capacity to discriminate *x* from *y*, then under normal circumstances S also has this capacity given that she has background knowledge that the target object is either *x* or *y*.

So far, we have focused on discrimination between two particular *objects* or two *persons*. Often we use our discriminatory capacities to discriminate one *kind* from another or one *type* from another, e.g. martins from blackbirds, gold from fool’s gold, or Ferraris from Lamborghinis. In analogy to the provided definition of paradigmatic discrimination between objects, we can define perfect discrimination between kinds and types as follows:

- **Perfect discrimination of kinds and types**
- S can perfectly discriminate Fs from Gs via M *iff* for every *x*
  1. in the nearest possible worlds where *x* is an F and where M is used, S believes via M that *x* is an F (and not a G) and
  2. in the nearest possible worlds where *x* is a G and where M is used, S believes via M that *x* is a G (and not an F).

For example, S can perfectly discriminate martins from blackbirds via eyesight iff (1) for every target object *x*, in the nearest possible worlds where *x* is a martin and where S uses eyesight, S believes via eyesight that *x* is a martin and (2), in the nearest possible worlds where *x* is a blackbird and where S uses eyesight, S believes via eyesight that *x* is a blackbird.

The two conditions on perfect discrimination are plausibly too strong for being necessary conditions on discriminatory capacities between kinds or types. In particular, we want to say that S has a general capacity to discriminate Fs from Gs even if there is one particular F-object (or if there are a few particular F-objects) that S takes to be a G-object. For example, there might exist one particular martin which S easily believes to be a blackbird via eyesight and still we want to say that S has the general capacity to discriminate martins from blackbirds via eyesight if S can discriminate all the other martins from blackbirds and all blackbirds from martins. Thus, for formulating plausible necessary and sufficient conditions for discrimination of kinds and types, we have to weaken conditions (1) and (2) as conditions for *most* (or *almost all*) Fs and most (or almost all) Gs. Since “most” and “almost all” are vague terms, this reformulation adds a further element of vagueness to the concept of discrimination beyond the vague concept of “nearest possible worlds.” However, this is not a problem but rather an advantage of the provided discrimination account since also the capacity of discrimination comes in degrees. S can have a better or worse capacity to discriminate Fs from Gs. Accordingly, the more Fs and Gs there are such that conditions (1) and (2) are fulfilled, the better S can discriminate between Fs and Gs.

## Conclusion

Actual instances of discrimination are manifestations of a discriminatory capacity. Discrimination does not allow for an internalist interpretation. Hence, an externalist theory is required. Reliabilist interpretations of discrimination face problems but modal interpretations are adequate. A version of sensitivity that takes particular alternatives into account and that is also stronger than ordinary sensitivity is necessary, and together with a corresponding adherence condition, also sufficient for discrimination. Safety in contrast is necessary but not sufficient for discrimination. The resulting modal theory of discrimination can be extended by distinguishing ordinary discrimination from reflective discrimination and from discrimination by having background knowledge. Moreover, a modal theory of discrimination cannot only be formulated for discrimination between objects, but also for discriminating between kinds and types.

## Footnotes

1 However, I regard self-sustained theories on widely neglected epistemic concepts as valuable partly because I believe in crucial conceptual differences between these concepts and knowledge.

2 Vogel (1987) and Sosa (1999) present cases of insensitive beliefs formed via induction that plausibly constitute knowledge, and Kripke (2011) presents implausible instances of closure failure for Nozick’s sensitivity account. For further discussions on sensitivity, see Becker and Black (2012).

3 For a criticism, see Blome-Tillmann (2015).

4 Moreover, relevant alternative theories sometimes rely on the concept of discrimination, at least implicitly. I will discuss the relation between relevant alternatives and discrimination in section two. Williamson (2013) thoroughly investigates the relation between identity and discrimination (or more precisely the relation between identity and indiscriminability) but he does not provide an account of discrimination in terms of necessary and sufficient conditions as it is done here. The theory of discrimination developed here is in line with Williamson’s account concerning central formal features of discrimination such as symmetry. However, it follows a different methodology. Knowledge-first epistemology takes the simple distinction between knowledge and ignorance as a starting point from which to explain other epistemic concepts. Accordingly, Williamson (2013) analyzes “discrimination between things as activation of the knowledge that they are distinct, and indiscriminability as the impossibility of activating such knowledge (p. 1).” This paper, in contrast, follows the methodology of developing self-contained theories of epistemic concepts other than knowledge and investigating the relations between these concepts and knowledge in a further step. For an application of this methodology for analyzing checking, see Melchior (2019).

5 For sure, we often ascribe a discriminatory capacity to someone without mentioning a particular method. However, in such cases we have a particular method in mind, for example observation, to which we can refer when asked. Moreover, one might suggest that we can acquire a general notion of discrimination simply by quantifying over methods, i.e. S has the general capacity to discriminate *x* from *y* just in case there is an M that fulfills certain conditions. However, such an account would be too weak and the capacity of discriminating *x* from *y* would become trivial. For example, everybody, or at least every adult, has the capacity to discriminate martins from blackbirds by asking an expert. Nevertheless, we do not want to say that every adult has a general capacity to discriminate martins from blackbirds.

6 Checking, in contrast to discriminating, plausibly requires intentionally using a method for determining whether *p* is true. See Melchior (2019).

7 See also Goldman (Goldman 1976, p. 780f.).

8 Williamson (2013) emphasizes that discrimination is symmetric. He also points out that indiscriminability is intransitive, i.e. it is possible that S fails to discriminate *x* from *y* and *y* from *z*, but still succeeds in discriminating *x* from *z*. Related issues concerning the relation between identity and discrimination will not be discussed in this paper.

9 Since I assume symmetricity of discrimination, I treat the formulations of ‘discriminating *x* from *y*’, ‘discriminating *y* from *x*’, ‘discriminating between *x* and *y*’, and ‘discriminating between *y* and *x*’ all as synonymous.

10 Such a modification does not have any impact on the central claims of the theory presented, which concern the modal conditions of discriminatory *capacities*, since the capacities of discrimination are not factive. Factivity only concerns actual manifestations of discriminatory capacities.

11 A general capacity to discriminate Fs from Gs via method M does not require that S can discriminate every F-object from G-objects. Plausibly, S can still discriminate Fs from Gs if there are some (but not too many) F-objects that S takes to be a G-object. We will discuss this issue in Sect. 4.

12 This does not mean that any belief results from exercising exactly one discriminatory capacity. If I form the belief that there is a computer in front of me, then this belief might result from my capacities to discriminate computers from desks, from chairs, from pencils, and so on.

13 There are various views on the market on which alternatives count as relevant. Most relevant alternative theories agree that the relevance of alternatives is in some sense context sensitive. Following DeRose (1992), we can distinguish two branches of relevant alternative theories. First, there are theories that claim that subject factors, i.e. factors of the believing subject, determine which alternatives are relevant. These subject factors are environmental factors of the believing subject according to one account or her practical interests according to subject sensitive invariantism. Goldman (1976) discusses the position that environmental factors are crucial but does not endorse it. For a version of subject sensitive invariantism that takes relevant alternatives into account, see Hawthorne (2004). Second, there are contextualist approaches that claim that attributor factors are crucial, i.e. factors of the knowledge attributor such as her practical interests. For versions of relevant alternatives contextualism, see Lewis (1996), Heller (1999), and Blome-Tillmann (2014).

14 The first example is due to Vogel (1990) while the second is due to Hawthorne (2004).

15 For the same reason, theories of discrimination and contrastivist knowledge accounts do not collapse into each other. Standard knowledge accounts accept that knowledge has a binary structure constituted by a subject S who knows proposition *p*. Contrastivism, as defended by Schaffer (2004a, 2005, 2007), has it that knowledge has a ternary, contrastivist structure, such that S knows *p* rather than a contrastive proposition *q*. For example, S knows that in the pen is a zebra rather than a monkey, but S does not know that in the pen is a zebra rather than a painted mule. However, S never knows that in the pen is a zebra simpliciter. Again, in cases like the zebra/painted mule case, discrimination and contrastivism fit together well since S can presumably discriminate zebras from monkeys but not from painted mules. However, in other cases such an analogy does not hold. I can know that my car is in lot 66 rather than in front of my house without knowing that it is in lot 66 rather than having been stolen five minutes ago although these cases cannot be plausibly explained in terms of discrimination (or lack of discrimination). The same holds for other lottery cases. Hence, contrastivist knowledge accounts plausibly cannot be spelled out in terms of discrimination.

16 See Goldman (1976) who develops a partial theory of perceptual knowledge based on discrimination.

17 At this point, one might object that these cases involve intellectual seemings. I do not find the concept of intellectual seemings convincing. Moreover, the method of discriminating odd numbers from even numbers does not involve any intellectual seemings. For a theory of intellectual seemings, see Chudnoff (2011). For a critical view on intellectual seemings, see Williamson (2007).

18 This formulation is meant to be short for: In the nearest possible worlds where the target object is *x* and where S uses M, S believes via M that it is *x* (and does not believe that it is *y*).

19 Nozick’s own terminology is not particularly clear. At one point, he argues that condition (3) guarantees that S’s belief is sensitive to the falsity of *p* whereas (4) ensures sensitivity to the truth of *p*. Here I will stick to the orthodox terminology that labels (3) the sensitivity condition and (4) the adherence condition. Nozick used subjunctive conditionals rather than possible worlds for expressing these two modal conditions, but for the sake of convenience, I will use possible worlds terminology. This terminological decision does not affect any of the following outcomes in this paper.

20 Nozick argues that S knows simpliciter if there is a dominant method M that outweighs the other methods, which fulfills the sensitivity condition and the adherence condition. However, these subtleties will not concern us here.

21 For a recent objection against the view that induction typically yields insensitive beliefs, see Wallbridge (2018). For a defense of Nozick’s sensitivity account against Kripke, see Adams and Clarke (2005).

22 For formulations of method-relative safety, see Pritchard (2005, 2007). It is disputable whether safety accounts of knowledge really avoid all the problems that they are designed to avoid. For example, safety also violates closure in Kripke’s case against sensitivity. See Murphy (2005).

23 Except for a kind of incomplete de re discrimination, as mentioned earlier.

24 Because this paper focuses on complete discrimination that leads to de dicto beliefs about the objects discriminated, this rules out the possibility that S can discriminate objects for which S does not possess a concept.

25 I make here some background assumptions about the rationality of S, in particular I assume that if S believes that the target object is *y*, S does not also believe that it is *x*.

26 Weak and strong versions cannot only be formulated for a-sensitivity but also for sensitivity simpliciter. Weak sensitivity: In the nearest possible worlds where *p* is false and where S uses M to determine whether *p* (or ~ *p*) is true, S does not believe via M that *p*. Strong sensitivity: In the nearest possible worlds where *p* is false and where S uses M to determine whether *p* (or ~ *p*) is true, S believes via M that ~ *p*. Nozick (1981) and his followers defend weak sensitivity.

27 One might suggest the following condition, which I label super-weak a-safety for *x* and *y*: In the nearest possible worlds where S believes via M that the target object is *x*, it is not *y*. If a target object is *x*, then it is not *y* but not vice versa. For this reason, super-weak a-safety is even weaker than weak a-safety. Presumably super-weak safety better captures the idea of discrimination between *x* and *y* than weak a-safety since it also takes S’s attitude towards an alternative *y* into account. However, we will see that weak a-safety is necessary but not sufficient for discrimination and that it does not capture the crucial feature of what it takes for a subject to have a discriminatory capacity. We trivially acquire the same results for super-weak a-safety, since it is weaker than weak a-safety. Therefore, I will hereinafter focus on weak a-safety.

28 Again, we can also distinguish a weak and a strong version of ordinary adherence and ordinary safety. Weak adherence: In the nearest possible worlds where *p* is true and where S uses M to determine whether *p* (or ~ *p*) is true, S does not believe via M that ~ *p*. Strong adherence: In the nearest possible worlds where *p* is true and where S uses M to determine whether *p* (or ~ *p*) is true, S believes via M that *p*. Notably, Nozick opts for a strong version of adherence but for a weak version of sensitivity. Weak safety: In the nearest possible worlds where S believes that *p* via M, *p* is true. Strong safety: In the nearest possible worlds where S does not believe via M that ~ *p*, *p* is true. Safety as defended in the literature is weak safety.

29 Note that opposing beliefs are different from using an opposing method that is known to be opposing. Suppose Peter knows that Paul can tell Anna and Hannah apart but that he thinks that Anna is named Hannah and vice versa. In this case, Peter will reasonably always believe the opposite of what Paul reports about the target person being Anna or being Hannah. In this case, Peter’s beliefs based on Paul’s testimony fulfill strong a-sensitivity and strong a-adherence.

30 There are two potential reasons for why a belief that *p* formed via M is safe. First, because S’s belief formation via M is properly responsive to the world. Second, because every ~ *p*-world is modally remote. In the latter case, safety is secured for purely modal reasons. Our beliefs that we are not brains in vats are safe for the second reason, as safety theorists argue. However, this purely modal possibility of safety fulfillment also raises problems for safety accounts. Hiller and Ram (2007) argue that safety can be fulfilled in Gettier cases for purely modal reasons and conclude that safety accounts of knowledge cannot solve Gettier problems.

31 In modal terms, we talk in a slightly different way about general capacities of discrimination and about actual instances of it. When talking about S’s capacity to discriminate *x* from *y*, we leave open whether S actually forms a true belief that the target object is *x* (or that is *y*). This is also left open in the formulation of conditions (D1) and (D2). In contrast, when talking about actual instances of discrimination, we sometimes presuppose that S truly beliefs that it is *x* (or that it is *y*) and, based on this presupposition, we argue that S’s true belief manifests a discriminatory capacity. Given this presupposition, we can correctly say that, in the nearest possible worlds where the object in question is *y instead of x* and S uses M, S believes via M that it is *y* (and not *x*). Counterfactual talk about an object being *y* instead of *x* conversationally implies that it is *x* in the actual world. These formulations cannot properly be used for characterizing general discriminatory capacities which are neutral with respect to whether the actual object is *x* or is *y*.

32 We can formulate a-sensitivity and a-adherence (strong and weak) for discrimination from the standpoint of *x* being the case and *y* being the alternative and vice versa. We acquire such an inverse formulation simply by exchanging *x* and *y* in the definitions. For example, here is strong a-adherence formulated for *x* (with respect to an alternative *y*), in brief, strong *xy*-adherence: In the nearest possible worlds where the object in question is *x* and where S uses M, S believes via M that it is *x*. However, this is precisely the definition of strong a-sensitivity for *y* with respect to *x*, i.e. for *yx*-sensitivity. We see that the formulation of strong *xy*-adherence is the same as the formulation of strong *yx*-sensitivity. We acquire, analogously, strong *yx*-adherence and strong *xy*-sensitivity simply by mutually exchanging ‘*x*’ for ‘*y*’. The same relations hold for *weak* a-sensitivity and weak a-adherence. We see how modal conditions of *xy*-sensitivity and *yx*-adherence and *yx*-sensitivity and *xy*-adherence can be reduced to each other. Analogous relations also hold for ordinary sensitivity and ordinary adherence with respect to *p* and ~ *p*. Strong ordinary adherence for believing that *p*: In the nearest possible worlds where *p* is true and where S uses M to determine whether *p* (or ~ *p*) is true, S believes via M that *p*. This is strong ordinary sensitivity for believing ~ *p*. Analogous reductions hold for weak ordinary sensitivity and weak ordinary adherence. Thus, there hold analogous reduction relations for ordinary sensitivity for *p* and ordinary adherence for ~ *p* as for a-sensitivity for *x and y* and a-adherence for *y and x*. However, these inversions do not play a crucial role for modal accounts of *knowledge*. For determining whether S knows that *p*, we formulate modal conditions such as sensitivity, adherence, and safety for beliefs in *p* and not for beliefs in ~ *p*. Hence, there is only one plausible label for these modal conditions.

33 Goldman (1976, p. 785f) reformulates his theory of perceptual knowledge in a sophisticated way in order to solve the dachshund problem. A theory of discrimination avoids this dachshund problem easily. S has the specific capacity to discriminate dachshunds from wolves but lacks the more general capacity to discriminate dogs from wolves. The aim of this paper is to formulate a self-contained theory of discrimination. As already noted, the implications for knowledge are not addressed in this paper.

34 However, I develop in Melchior (2019) also an account for checking whether *p* and not a particular alternative *q* is true. These instances of checking require a-sensitivity, but they also involve intentionally using a method while discrimination does not.

35 Developing such a checking-based theory of proof in court is beyond the scope of this paper. Therefore, this claim is here formulated only as a conditional. Moreover, I will not discuss here alternative explanations of why proof in court might be sensitive.

36 One could add here a-adherence, also a necessary condition for discrimination. However, we have seen that any version of a-adherence is also a version of a-sensitivity.

37 Bishop (2010) argues that all plausible theories of justification, not only externalist ones, suffers from the generality problem.

38 For the formulation of the generality problem, see Conee and Feldman (1998). For specifying methods in the context of sensitivity, see Becker (2012).

39 For acquiring a complete description of one’s discriminatory capacity, we not only have to take environmental conditions but also subject-dependent conditions into account, e.g. whether S is sober or drunk.

40 Checking, as I propose in Melchior (2019), does not suffer from the generality problem because it always involves intentionally using a method and these intentions determine the respective method.

41 Even Nozick uses his concept of knowing via a particular method only as a first step for analyzing knowing simpliciter. In a second step, he argues that S knows that *p* iff there is a method which fulfills the adherence condition and the sensitivity condition and that modally dominates (or in his terminology outweighs) the other methods.

42 For the same reason, this circumvention of the generality problem for discrimination is not applicable to a modal concept of undermining, which does not refer to a particular method.

43 I assume here that knowing that one has a discriminatory capacity requires that one knows that particular methods have certain modal features. I do not mean that the subject must explicitly conceptualize these beliefs in terms of modal conditions. Such a requirement would be too demanding. Rather, it is only required that the subject holds certain (implicit) beliefs in terms of counterfactual conditionals. However, one might accept a purely externalist account of reflective knowledge that only requires that one’s belief that one has a discriminatory capacity is itself reliable or safe. I take it that the suggested notion of reflective discrimination better captures the idea of a more reflective concept of discrimination, which also includes the ability to defend the view that one has a discriminatory capacity against objections. However, different concepts of reflective discrimination can be formulated.
